# EEG biomarkers of Epileptiform Activity in Relation to Alzheimer’s Disease Biomarkers

**DOI:** 10.1002/alz.088969

**Published:** 2025-01-09

**Authors:** Andras Attila Horvath

**Affiliations:** ^1^ National Institute of Mental Health, Neurology and Neurosurgery, Budapest Hungary

## Abstract

**Background:**

Growing evidence suggests that the imbalance between excitability and inhibitory neural activity is a key aspect of cognitive decline. Subclinical epileptiform activity (SEA) has been indicated as a marker of increased cortical excitability. While SEA is considered as a benign EEG sign in the elderly population, recent studies demonstrated its role in the progression of Alzheimer’s disease. The aim of our study was to investigate the relevance and phenotype of SEA in clinically healthy elderly population.

**Method:**

We studied 62 elderly subjects determined as healthy based on physical, neuropsychology, neuroimaging (MRI), and serological workup. All subjects underwent 24‐h Holter electroencephalography (EEG), visual analysis was conducted by two independent raters. Subjects were classified into EEG positive (EEG+) and negative (EEG‐) groups based on detecting epileptiform activity on their EEG. Neuropsychology battery and structural MRI results were compared between the groups.

**Results:**

EEG analysis showed epileptiform activity in 26% of cases. EEG positivity was associated with significantly lower verbal and category fluency subscores on Addenbrooke Cognitive Examination (p=0,03 and p=0,01). This group also performed worse on both Trail Making Test A and B (p =0,02 and 0,003 respectively). Age and education had a significant effect on neuropsychological performance in both groups. Structural MR analysis showed significant group differences in various areas, particularly in the parietotemporal regions (p=0,001). Interestingly, EEG+ patients had larger external temporal brain volume bilaterally (p<0,001) and reduced medial parietal volume (p=0,001).

**Conclusion:**

Presence of SEA among healthy elderly shows a discrete but significant association with decreased cognitive performance in certain subdomains, as well as a special imaging phenotype. Our results underscore the relevance of data suggesting that cortical hyperexcitability associate with parietal atrophy and preserved temporal structures. In Alzheimer’s disease, the detailed representation corresponds to the hippocampal sparing phenotype. Considering our findings, SEA might associate with an increased risk for cognitive decline even in healthy individuals.

